# Estimating Historical Eastern North Pacific Blue Whale Catches Using Spatial Calling Patterns

**DOI:** 10.1371/journal.pone.0098974

**Published:** 2014-06-03

**Authors:** Cole C. Monnahan, Trevor A. Branch, Kathleen M. Stafford, Yulia V. Ivashchenko, Erin M. Oleson

**Affiliations:** 1 Quantitative Ecology and Resource Management, University of Washington, Seattle, Washington, United States of America; 2 School of Aquatic and Fishery Sciences, University of Washington, Seattle, Washington, United States of America; 3 Applied Physics Lab, University of Washington, Seattle, Washington, United States of America; 4 National Marine Mammal Laboratory, Alaska Fisheries Science Center, Seattle, Washington, United States of America; 5 School of Environmental Science and Management, Southern Cross University, Lismore, New South Wales, Australia; 6 Protected Species Division, Pacific Islands Fisheries Science Center, Honolulu, Hawaii, United States of America; Universite Paris Sud, France

## Abstract

Blue whales (*Balaenoptera musculus*) were exploited extensively around the world and remain endangered. In the North Pacific their population structure is unclear and current status unknown, with the exception of a well-studied eastern North Pacific (ENP) population. Despite existing abundance estimates for the ENP population, it is difficult to estimate pre-exploitation abundance levels and gauge their recovery because historical catches of the ENP population are difficult to separate from catches of other populations in the North Pacific. We collated previously unreported Soviet catches and combined these with known catches to form the most current estimates of North Pacific blue whale catches. We split these conflated catches using recorded acoustic calls from throughout the North Pacific, the knowledge that the ENP population produces a different call than blue whales in the western North Pacific (WNP). The catches were split by estimating spatiotemporal occurrence of blue whales with generalized additive models fitted to acoustic call patterns, which predict the probability a catch belonged to the ENP population based on the proportion of calls of each population recorded by latitude, longitude, and month. When applied to the conflated historical catches, which totaled 9,773, we estimate that ENP blue whale catches totaled 3,411 (95% range 2,593 to 4,114) from 1905–1971, and amounted to 35% (95% range 27% to 42%) of all catches in the North Pacific. Thus most catches in the North Pacific were for WNP blue whales, totaling 6,362 (95% range 5,659 to 7,180). The uncertainty in the acoustic data influence the results substantially more than uncertainty in catch locations and dates, but the results are fairly insensitive to the ecological assumptions made in the analysis. The results of this study provide information for future studies investigating the recovery of these populations and the impact of continuing and future sources of anthropogenic mortality.

## Introduction

The blue whale (*Balaenoptera musculus*) is an endangered species with three widely recognized subspecies found in the Southern Hemisphere (*B.m. intermedia*), Indian Ocean (*B.m. brevicauda*) and in the North Pacific and North Atlantic (*B.m. musculus*) [1], [2]. Many populations are heavily depleted due to targeted whaling through their distribution. In the North Pacific, blue whales were hunted from 1905–1971 and despite decades without commercial catches the status of their recovery is unknown [3]. At present a large population known as the eastern North Pacific (ENP) population feeds each summer within 30 miles of the California coast and is believed to range from the equator to the Gulf of Alaska [4]. The ENP population is the one of the most accessible blue whale populations in the world and has been the focus of many studies, such as feeding mechanics and behavior [5], behavioral ecology [6] and responses to anthropogenic noise [7]. However, a formal assessment of their current abundance relative to historical levels has not been conducted, largely because of the difficulty in separating historical catches of the ENP population from catches from other populations in the western North Pacific (WNP).

**Figure 1:**
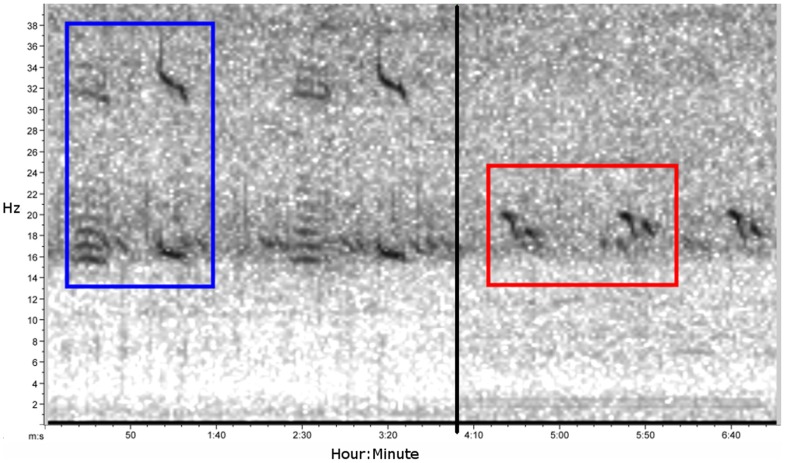
Spectrogram showing the ENP and WNP blue whale song calls. These calls were recorded on a hydrophone in the Gulf of Alaska at different times; the blue box shows the ENP song call and the red box the WNP song call. The clear distinction between the two is used to differentiate the presence and absence of the two populations.

A key step in separating catches is using vocalization occurrence data to estimate the migration pathways of the ENP and WNP populations over time and space. Analyses of these vocalizations observe two distinct NP blue whale ‘song call’ types (Figure 1) [4], [8]–[10], which are assumed to be produced by the ENP and WNP populations. The ENP song call is comprised of the rhythmic repetition of a two part vocalization known as the ‘AB call’ type [11]. The AB call type components are produced exclusively by males in a variety of behavioral states [6]. The AB song calls are observed only in lone, traveling males and are produced year-round and thus likely have some kind of reproductive function, although their exact purpose is unknown [6]. The WNP song call is a single part call repeated as song in a similar way as the AB song of the ENP population, but has a clearly identifiable and distinct form (Figure 1) [8]. No studies have examined the behavioral context of WNP song, but it is assumed to be similar in function to the ENP song. Another common call type observed in the ENP population (and other populations of blue whales) is the downswept ‘D’ produced by foraging groups of both sexes [6]. A recent study found a temporal separation in the production of the AB and D call types at a summer feeding area, and argued that both were necessary for an accurate assessment of the timing of fine-scale seasonal movements into foraging regions [12]. Unfortunately there is no evidence of population differences in D-like calls as there is with song calls, and so we focused exclusively on the song call occurrence patterns.

Besides distinct song call types, the acoustic analyses also show that broad spatial migration patterns over time can be qualitatively distinguished from the data: the ENP population inhabits waters from the Costa Rica Dome to the Gulf of Alaska (GOA) and the WNP population is heard predominantly between Kamchatka and the GOA. We use the timing of occurrence of these song calls of the two populations to quantitatively model the seasonal movements by the two populations.

Additional data sources beyond vocalization differences support the hypothesis of two populations of blue whales in the North Pacific. Satellite tags, photographic identification, and sightings showed individuals moved between the Gulf of Alaska, the Californian coast, and the eastern tropical Pacific [13], [14], although such data are not available for the WNP population. In addition, there are significant differences in length between whales in the ENP and WNP [15]. Finally, Gulf of California blue whales are genetically similar to those off California [16], although there are no studies investigating genetic differences between the ENP and WNP populations. Taken together there is substantial evidence that the ENP population is separate from blue whales in the WNP, which we used to validate the acoustics-based modeling approach taken in this study. While diverse sources of evidence point toward a single ENP population, the population structure of the central and western NP remains unclear, and there may be additional populations in these regions. For example, there is some speculation that a population off coastal Japan was extirpated and that another exists around Hawaii [17]. The acoustic and other data shed little light on these hypotheses, which do not affect our estimates of ENP catches, although we recognize that what we call the ‘WNP population’ may include additional population structure.

The recovery and resilience of the ENP population are unknown [3], but can be estimated using abundance estimates combined with historical catches [18]. Abundance estimates are available for the ENP population [19], [20], but historical catches have not been split between ENP and WNP populations because blue whale population structure was unknown during commercial whaling and catches were reported only as blue whales. We estimated the probability of each catch belonging to the ENP population to separate the conflated catch series and produce an ENP and WNP catch series that can be used in future assessments. Qualitatively, it was clear from the acoustic data that whales caught off Japan and Kamchatka are highly unlikely to be from the ENP, and likewise blue whales caught off California were likely to be from the ENP population. However, splitting catches in the GOA is more complicated because the two populations overlap in space and time there during the feeding season [9].

In this study we collated the most up-to-date time series of historical catches of blue whales in the North Pacific, including previously unreported Soviet catches in the 1970s. Using song call occurrence data, we modeled the probability that a whale caught in a particular location and month belonged to the ENP population, and applied this model to the historical catches. The resulting time series of catches for ENP and WNP blue whales are the first to be obtained objectively for each population.

## Materials and Methods

### Catch Data

Official catches for NP blue whales were obtained from two databases maintained by the International Whaling Commission (IWC): the ‘summary database’ [21] and the ‘catch database’ [22]. For both databases, the expedition code referred to either a pelagic fleet operating in a predefined IWC region, or catches taken back to a land station for shore-based processing; thus a pelagic vessel operating in multiple regions in the same year would be recorded as different expeditions. For most expeditions the start and end month of each operation were recorded annually.

The summary database contained annual catch totals by species (including blue whales) for each whaling expedition since 1900, but contained no detailed information about the exact location or date of individual catches. Catches with unspecified species accounted for 8,519 out of 570,146 (1.5%) total whales of all species in the North Pacific. We estimated how many unspecified catches were likely to be blue whales and included them in the analysis as follows: (1) For expeditions with no catches identified to species in a year, we used the proportion of blue whale catches relative to all other species in adjacent years and applied that to the number of unspecified catches. (2) For expeditions with unspecified catches and catches reported to species in the same year, we used the proportion of blue whales within that year. (3) For the substantial unspecified catches (4,415 whales) from coastal Japan after blue whaling started (1905–1909), we assumed blue whales were 16.4% of all whales. This was the average of the following two years (1910–1911), after which the proportion of blue whales caught off coastal Japan declined quickly. Using these methods we added 49 blue whale catches off British Columbia (1907 and 1914), 81 off the US west coast (1918–1938) and 782 off coastal Japan (1905–1909 and 1934–1936).

**Figure 2:**
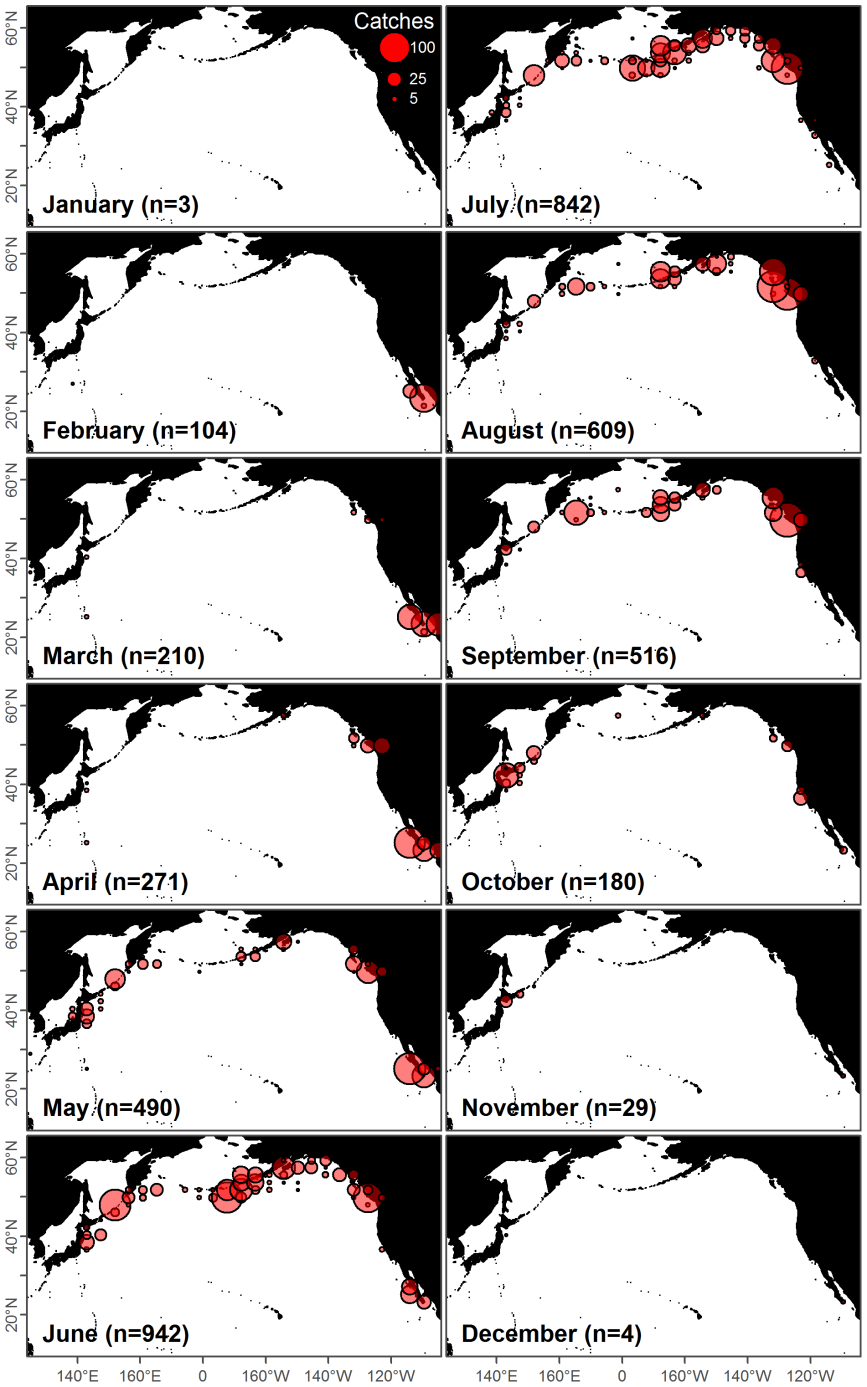
Distribution of the blue whale catches with reported locations. Monthly maps showing blue whale catch locations that were reported without uncertainty (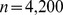; 43% total) to the International Whaling Commission. There are clear spatial and temporal patterns reflecting the migrations of the two populations and whaling seasons.

The catch database contained data for individual whales including species, location, date, expedition, length, length of fetus (if present), and sex (Figure 2). Detailed information was not available for all catches, particularly in earlier years and during the period of later Soviet whaling, thus the catch database contained only a subset of the summary database.

These databases contained all catches known to the IWC as of November 2011. However, it has been known since the mid-1990s that the USSR caught additional blue whales in the 1960s and 1970s that were misreported or not reported to the Bureau of International Whaling Statistics and are missing from the IWC databases [23]. These discrepancies were recently resolved for catches in the North Pacific with recovered original scientific reports, adding 738 (7.6% of total) blue whale catches to the previous version of the IWC annual database [24]. In many cases the Soviet reports gave clues about the likely date and locations of these catches, which were used to help infer likely catch locations. Not all of these reports were available and uncertainty remained surrounding the number, locations and dates of some Soviet catches during 1962–1971 [24]. We accounted for this uncertainty explicitly in the analysis as described in the next section. Outside of these years few, if any, blue whale catches were missing.

### Catch Uncertainty

In addition to missing data about Soviet catches, many other catches in the IWC catch databases are missing locations, dates or both (Table 1), and the original records are lost to history. This ‘catch uncertainty’ must be explicitly taken into account when separating catches to reliably quantify the uncertainty associated with the catch time series. We used a Monte Carlo method to integrate over all potential locations and months for each individual catch. Conceptually, possible sets of locations and months were determined for each catch, from which random samples could be generated. By generating and splitting many potential catch series, the catch uncertainty was propagated through into the uncertainty in the final results. The challenging part was to determine accurate sets of locations from which to draw randomly. We assigned catches into five categories ranging from the most uncertain location (IWC Region) to the most certain (exact position known), and treated each category as described below.

**Table 1:** North Pacific blue whale catches by categories of uncertainty.

Location Uncertainty Category	Uncertain Month (%)	Certain Month (%)	Total (%)	Category Description
IWC Region	3977 (41%)	1 (0%)	3978 (41%)	Known to be within a large rectangular region (Figure 3)
Partial Locations	447 (5%)	477 (5%)	924 (9%)	Catches of other species by the same expedition and year are available.
Inferred	447 (5%)	0 (0%)	447 (5%)	Able to infer accurate locations with high confidence.
Soviet	108 (1%)	116 (1%)	224 (2%)	Original Soviet whaling logs give information about locations and months.
Certain	0 (0%)	4200 (43%)	4200 (43%)	Reported to the IWC.
Total	4979 (51%)	4794 (49%)	9773 (100%)	

The percent of all catches is indicated in parentheses after the number of catches. Some exact catch positions and dates are unavailable and were inferred with varying levels of uncertainty for many cases.

**Figure 3:**
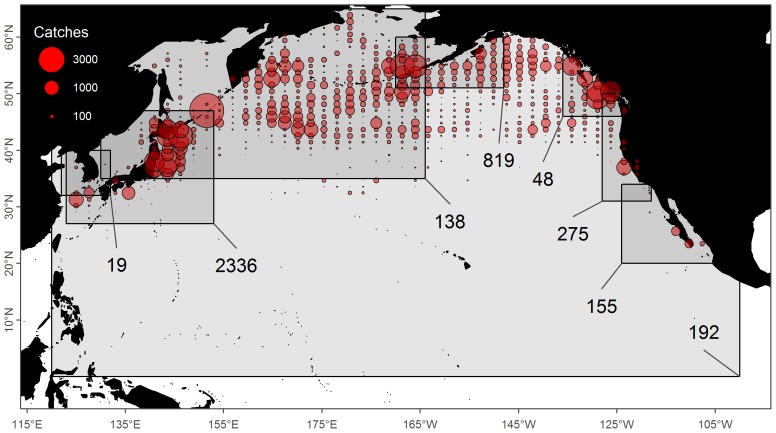
Map of reported blue, fin, sei and common minke whale catch locations (1905–1971). These catch locations were used to infer potential locations for blue whale catches without known locations. 41% of catches are known only to broad, management-defined regions (shaded boxes) which are shown with their corresponding blue whale catches.

(1) ‘IWC Region’ (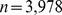 catches) was the most uncertain category and only occurred when no location information was available for an expedition in a year beyond the broad regions defined by the IWC. IWC regions are defined by longitude and latitude ranges (i.e. a rectangle, Figure 3), which cover both land as well as regions of ocean where no whaling ever occurred. Clearly, randomly drawing uniform locations from these large regions would lead to inaccurate and even nonsensical catch locations. Instead, we drew from locations within each IWC region where catches of blue whales and other species had actually been taken. There was too little variation in reported blue whale catch locations (Figure 2), while conversely some species such as sperm (*Physeter macrocephalus*), humpback (*Megaptera novaeangliae*) and bowhead (*Balaena mysticetus*) whales occupy very different habitations than blue whales. Therefore we randomly drew from the reported locations of catches of known locations of blue whales and other species most similar to the blue whales: blue (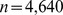), fin (*B. physalus*, 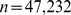), sei (*B. borealis*, 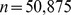), and common minke (*B. acutorostrata*, 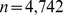) that were caught during 1905–1971 when blue whales were targeted. By drawing from actual catch locations from similar species, we accounted for spatial patterns of whaling effort, but implicitly assumed this effort was similar between species and that blue whales occupied the same spatial extent as other species. This assumption generally increased the spatial uncertainty of catches compared to the scenario where we randomly drew only from locations where blue whales were caught. Although catches in the IWC region category are the most uncertain, most of these catches came from Japanese and Korean waters (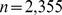) and Aleutian islands (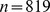) which likely contain mostly WNP blue whales (Figure 3).

(2) The ‘Partial Locations’ category (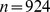) was where an expedition reported locations for some species but not for some blue whales. Most of these catches (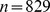) were from Soviet pelagic expeditions where the month is known and can be cross referenced with the catch database. However, there were a few cases (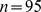) when an expedition reported some, but not all, of the catches in a year. We inferred locations of catches in this category by drawing, with replacement, from locations of all species in the same month.

(3) The ‘Inferred’ category (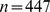) was where the location was not reported, but could be inferred with high precision. Most often this occurred when catches were reported as coming from a land station with a known location in a previous year, but no location reported in the current year. The unreported catches must have the same locations as previous years since the land stations were stationary. There were some catches (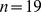) with unknown locations but known dates, in addition to known locations of catches of other species on the same dates. For these cases averages of locations of adjacent catches of other species were used without uncertainty.

(4) The ‘Soviet’ category (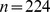) was specifically for Soviet catches from 1962-1971 that were updated in [24] and whose original reports allowed us to determine smaller rectangles than the broad IWC regions. We assumed the vessels could have been anywhere within these rectangles and therefore drew random uniform locations from each rectangle.

(5) The ‘Certain’ category (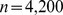) was for catches where the exact location was reported in the catch database. These are, fortunately, the most common case (43% of the total), and we did not consider any uncertainty in these locations.

Blue whale catch dates were also often not reported (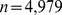, 51%) in the IWC catch database. We applied the same Monte Carlo approach to account for uncertainty in date by sampling from the possible months. We used an expedition's operating period from the IWC summary database to determine the range of possible months. If the operating period for an expedition was missing, we assumed the catches could have been from any month. However, we assigned a probability to each potential month that reflects the fact that most blue whales were caught in summer because of defined whaling seasons, weather limitations, and seasonal availability of whales. This weighting was based on the proportions of whales caught in each month for the same set of species used for geographic uncertainty (blue, fin, sei and common minke whales).

Thus, there was a range of potential locations and dates for each catch depending on the level of detail reported (Table 1). A routine was written in the statistical framework R [25] which randomly samples from the sets of locations and months for all catches, and returns a dataset which represents a possible realization of the conflated catch series. Repeatedly running this routine to generate sets of potential catch series (i.e. a Monte Carlo approach) allowed us to include catch uncertainty in our estimates of ENP catches. This approach assumed the unknown catches were not systematically biased in space and time compared to the known catches. The generated sets of conflated catch series were then propagated through to the process of assigning catches to populations.

### Acoustic Data

**Figure 4:**
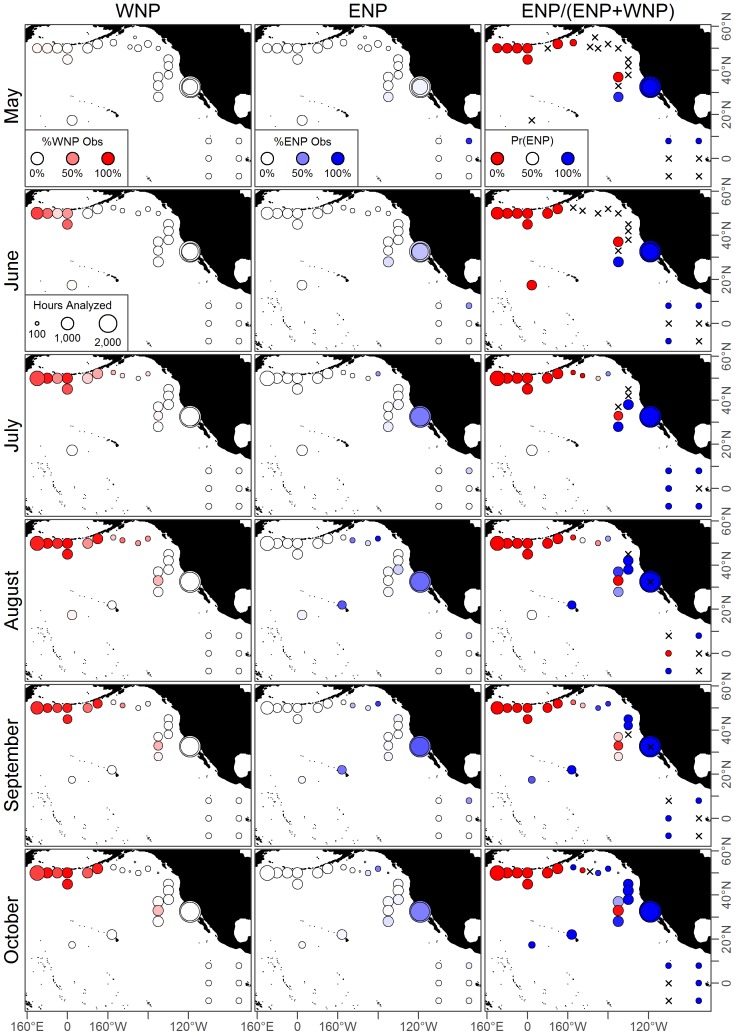
Observed hydrophone data for May-October. These data are used to fit separate acoustic models for both populations. Colored circles show hydrophones and the proportion of hours calls were observed while and “x” denotes those with no observed calls for either population. The western population migrates east along the Aleutian Islands and the eastern population migrations northwest into the North Pacific. Hydrophones with observed calls from both populations clearly show overlap for much of the North Pacific.

**Figure 5:**
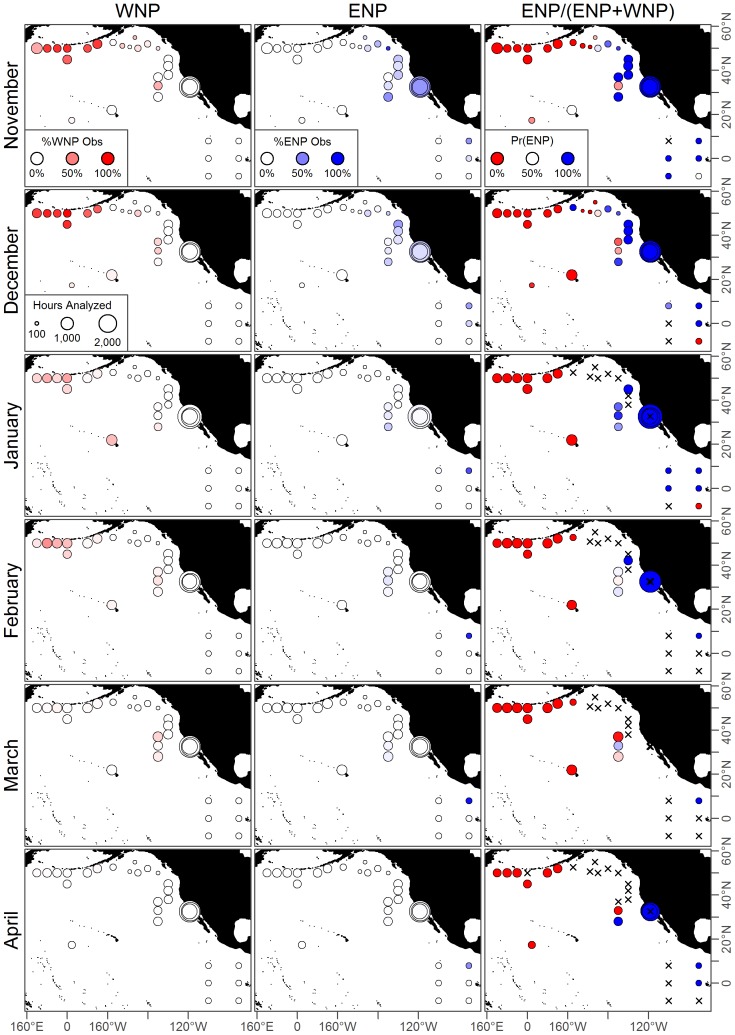
Observed hydrophone data for November-April. Caption as for Figure 4.

Detections of ENP and WNP song calls came from six sources in the North Pacific (Table 2, Figures 4 and 5). For all hydrophones, data were converted into hourly presence or absence of ENP and WNP calls. Hydrophone sources were: (1) six hydrophones placed by the National Oceanic and Atmospheric Administration (NOAA) in the eastern tropical Pacific [26]; (2) sixteen US Navy Sound Surveillance System and other hydrophones spread across the North Pacific; note that their exact locations were classified and approximated based on previously published studies [8]; (3) six hydrophones in the Gulf of Alaska placed by NOAA - after 9 months, one of these hydrophones was discontinued and another was moved slightly [9]; (4) six hydrophones in the Channel Islands and Cortez and Tanner Banks deployed by Scripps Institution of Oceanography [12]; (5) one hydrophone moored off Oahu, Hawaii, described in [27] and used in [8]; and (6) a single hydrophone off Wake Island [8].

**Table 2:** Summary of the groups of acoustic hydrophones used in this study.

Group	# Hydrophones	Begin Date	End Date	Reference	Notes
ETP^1^	6	May-96	May-97	[4]	N/A
GOA^2^	6	Oct-99	Jun-01	[9]	One hydrophone removed after 5/2000, another moved in 5/2000
SOSUS^3^	13	Nov-95	Nov-96	[8]	Hydrophone positions estimated as they are still ‘protected’
Wake	1	Apr-92	Dec-92	[8]	Missing months
Kaneohe	1	Aug-92	Apr-93	[8], [27]	Missing months
CH^4^	6	Aug-00	Feb-04	[12]	Down during 5 February to 15 April 2002

1 Eastern Tropical Pacific;

2 Gulf of Alaska;

3 US Navy Sound Surveillance System;

4 Channel Islands.

The GOA data are a random sample of 15% of total hours, while the rest are 100% of total hours. Several hydrophones were inoperable for periods of time due to hardware failures, leading to gaps in the data. There is little overlap in year between the groups. See specific references for further details of the data.

Presence was recorded when at least one call was detected in an hour of analyzed data, implying no differentiation in the number of calls within an hour. The presence data were then aggregated by month, and across years for the GOA and Channel Island hydrophones. Specific details regarding hydrophone deployment and methods for processing the raw data can be found in the original references (Table 2). Taken together these 32 hydrophones covered a large expanse of the North Pacific (Figures 4–5), and provided a unique opportunity to model the large movement patterns undertaken by both populations of blue whales.

### Acoustic Presence Models

The acoustic data used here contained information about NP blue whales' distribution both in space and season, from which migration patterns could be inferred for the two populations. We used these patterns to infer to which population the conflated historical catches belonged. We developed a two-stage model that (1) estimated the presence or absence of each population in space and time and then (2) predicted the probability a catch belonged to the ENP population. The first stage fitted a spatial surface separately to the ENP and WNP populations based on the monthly call rates. These two independent ‘acoustic models’ predicted the probability of observing at least one song call in an hour at a given location and time. In the second stage the two acoustic models were combined together into a single ‘prediction model’ used specifically for predicting the probability that a conflated catch belongs to the ENP population, given its location and month. This section describes the motivation and development of the acoustic models and how they are combined into the prediction model.

To develop these acoustic models we assumed the spatiotemporal song call patterns observed in the data reflected an underlying occurrence and used the data to estimate this occurrence. The first choice in developing the acoustic models was deciding which likelihood function is most appropriate for the data. Since the data were presence-absence (a whale either was or was not detected in each hour), a binomial likelihood seemed like a natural choice. However, sequential hours of detection at a location may not have been independent, as is expected in a true binomial experiment, since a single traveling or resting whale will potentially be recorded calling for many consecutive hours. If a call was heard in one hour, it would likely be heard there in the following hour as well. As a result, the variance in the data was expected to be higher than a binomial process. In other words, the data were over-dispersed. We addressed this issue by adding an over-dispersion parameter to the binomial model to increase the expected variance, which had the effect of turning the binomial model into a beta-binomial model. The parameterization of the beta-binomial used in this study had a likelihood function of 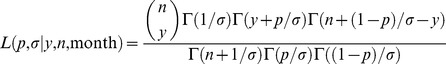
where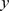 was the number of hours with at least one observed call, 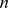
the total hours analyzed, 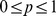 the probability of observing at least one song call in an hour, 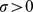 the over-dispersion term, and 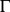 is the gamma function. For this distribution, 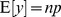 and 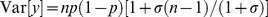. As 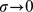, the variance becomes 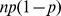, which is the variance of a binomial model, demonstrating that the 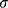 parameter increases the variance as desired. The support in the data for a 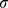 term in the model was tested in the model selection phase.

In developing the acoustic models we used only covariates which were also available for the catches. Thus, while oceanographic conditions at the hydrophones likely could have explained the occurrence of blue whales [28], we could not obtain these oceanographic data for the historical catches. We therefore included only latitude, longitude, and date as covariates in the model, and the implications of this assumption were explored in the ‘Ecological Uncertainty’ section below. The positions were used as is, but it was natural to aggregate time into appropriately-sized units. We chose to bin time into months because anything longer in duration may have missed the fine-scale movements, and the weeks-long delay between presence and singing found in [12] precluded anything shorter.

The relationship between occurrence and spatial positions is complex, and unlikely to follow a smooth mathematical form (e.g. linear, quadratic, etc.). We therefore used nonparametric models to infer the relationship between explanatory and response variables. Typically the non-normal sampling and nonparametric nature proposed here would have implied a generalized additive model (GAM). However, GAMs are not flexible enough to accommodate beta-binomial sampling [29]. We therefore used a more flexible modeling platform called generalized additive models for location, shape and scale (GAMLSS) which was developed to extend GAM models to handle more complex distributions, including the beta-binomial [30]. The GAMLSS package in R [25], [30] allowed additive predictors on both parameters of the beta-binomial distribution simultaneously, so that both varied spatially in a nonparametric way. We chose additive position terms and categorical month terms for both parameters as the most complex case (i.e. the full model) and fit, for example, the ENP model as:



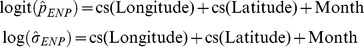
where 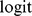 and 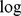
were the canonical link functions and 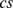
is a cubic spline smoother. The same structure was also used to fit the full model for the WNP data. The resulting acoustic models predicted the probability of observing at least one song call in an hour for a given position and month.

Model selection proceeded by fitting several plausible models and comparing corrected Akaike information criterion (AICc) values [31], as well as examining model residuals using standard diagnostics. Model selection was not used to infer biologically meaningful predictors of blue whale habitat, as was done for example in [28], but rather to identify the most appropriate structure and complexity of the models of blue whale song call occurrence. To test the statistical support for over-dispersion we fitted both binomial and beta-binomial GAMLSS models. Likewise, to test the additive nature of the models, a beta-binomial model with linear terms was also considered. Three variants of the beta-binomial GAMLSS model were also fit, having either constant, linear, or additive terms to investigate the spatial complexity of over-dispersion. The best supported acoustic models for the ENP and WNP represent the first of our two-stage model.

### Prediction Model

The second stage of the modeling was to develop a model which predicts the probability that a catch belonged to the ENP given its location and month. To make these predictions we combined the ENP and WNP acoustic models together. Conceptually, if the ENP acoustic model predicts high occurrence in a region and the WNP model does not, we would assign catches in that region to the ENP population. Specifically if we let 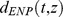 and 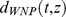 be the density of whales at date 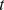 and location 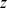 then the probability a catch belonged to the ENP is: 
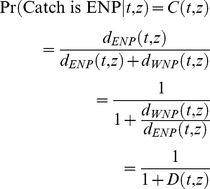



Clearly the densities of the populations dropped during the period of historical whaling. However, the way the ratio of the densities, 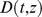, changed over years is unclear because the abundance trends of both populations are poorly understood. For our base case we therefore assumed that both populations were depleted at an equal rate so 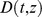 was independent of year and simplified to 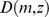 for month 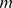. We further assumed the ratio of densities was approximately proportional to the ratio of the probabilities of call occurrence:



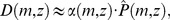



Where 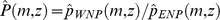 is the ratio of estimated call probabilities and 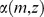 is an unknown scaling factor that may change over month or space in response to many external influences. Lacking any information on its true value, we assumed a default of 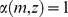 for the base case and the sensitivity to this assumption was explored below. Thus the base case prediction model is



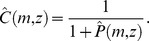



The above formula combined the two acoustic models together into a prediction model that can be applied directly to the catches.

The GAMLSS models provide standard error estimates only at the positions of the hydrophones, but the predictions were to be interpolated and extrapolated, sometimes widely, to predict the catches. Since accurately quantifying the uncertainty in predictions is an important aspect of modeling these data, we used a common alternative approach known as nonparametric bootstrapping [32]. With this approach the acoustic and prediction models were refit to sets of acoustic data resampled with replacement. For each resampled set of data, the acoustic models were refit, and the predictions of ENP catches were recalculated. Since the Monte Carlo method for quantifying uncertainty in catches also relied on sampling repeatedly to account for uncertainty over potential positions and months, it was natural to incorporate the two resampling steps together. The algorithm used to accomplish this was:

1. Generate a random realization of a potential conflated catch series 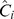.
2. Bootstrap the acoustic data by sampling with replacement from rows of the acoustic data. Each row contained the proportion of hours with presence in a month for a hydrophone.
3. Refit the two GAMLSS acoustic models to the bootstrapped acoustic data.
4. Use the fitted bootstrapped models to predict an ENP catch series 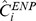.
5. Aggregate the split catches by total and year to create time series.
6. Repeat steps 1-5 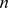 times.
7. Summarize the results using the median and percentiles over the set 
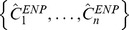
.

This algorithm simultaneously incorporated both the catch and statistical uncertainty in allocating catches between the two populations and thus made it difficult to compare the relative sizes of these uncertainties. We therefore also ran the algorithm without step 3, in essence removing the effect of bootstrapping so that only catch uncertainty was estimated. Implementing this algorithm split the catches and quantified the relative size of the uncertainties.

### Ecological Uncertainty

There was one more important source of uncertainty that affected the results. In the base case results we made two big assumptions in setting 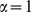: (1) the ratio of densities at a location was identical across years and equal to the ratio of call probabilities and (2) the behavioral context of song calls were identical over time and space, and between populations. By fixing 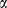 we implicitly ignored the potential uncertainty in these assumptions and their impact on the final results. Deviations from these ecological assumptions could arise from a variety of biological, environmental or anthropogenic factors. Examples of potential biological factors are different calling rates between populations, dynamic migration patterns (over year, space, or seasons) due to changing environmental conditions, and differences in the behavioral context of singing between populations. The largest potential anthropogenic factor was if whaling depleted the two populations at different rates so that the ratio of densities of the populations changed over time.

The impact of misspecifying 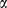 on the results depends on the size of 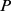: in regions where only one population is found the impact will be small, and the impact is highest when the populations overlap the most. Thus the ‘ecological uncertainty’ caused by using an incorrect 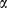 value varies spatially with the estimate of the ratio of call probabilities. Consider the example where the truth is a prediction model with 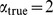, then following cases illustrate how assuming 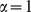 impacts predictions of catches. If 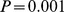 for some location, as would occur in predominantly ENP regions, then 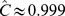 instead of the true value 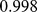 and likewise if 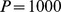, as in predominantly WNP regions, then 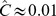 instead of 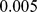. Thus for extreme values of 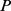 the effect of misspecifying 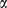 is mitigated by the nature of the structure of the prediction model. However, if 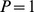 then 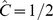 instead of 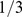, so the impact of ecological uncertainty is more pronounced in regions where both populations are singing at the same rates. Due to the complex relationship between 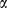 and the ratios of densities and call probabilities it is difficult to quantify and interpret 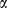 in terms of different biological and anthropogenic scenarios.

The veracity of the ecological assumptions about 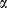 could not be tested against data, but there was no reason to expect it would be constant across space for almost a century. We therefore conducted a sensitivity test where we assumed that 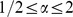 and that a randomly drawn 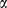 was constant across all catches within an iteration of our algorithm. Values were randomly chosen from a uniform distribution on the log scale so that 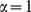 was equally likely to be low or high.

### Model Validation

We used information from previous studies to corroborate the catch splitting model developed here. The current distribution of ENP blue whales based on satellite tag and photographic re-sighting studies were compared to our model for ENP whale distribution as a qualitative check.

In addition, we used differences in mean total lengths to quantitatively validate the model. A previous study [15] found that WNP blue whales were longer than ENP blue whales, therefore whales with a low probability of being ENP should be longer than those with a high probability. We used the same selection criteria for mature females as in [15] for our length validation analysis, and performed two statistical analyses: a two-sample 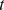-test comparing mean length differences by population, and a linear regression of model predictions against length. For the 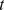-test we assigned catches to the ENP if their predicted probability was greater than 0.5, and WNP otherwise. Both statistical tests were performed for all 1000 bootstrapped sets of predictions, so that there was a distribution of mean length differences, regression lines, and 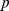-values for both tests. If our model is correct, we should find that blue whales assigned to the ENP population are significantly shorter, and there should be a significant negative relationship between length and the predicted probability of being an ENP blue whale.

## Results

### Acoustic Presence Models

**Figure 6:**
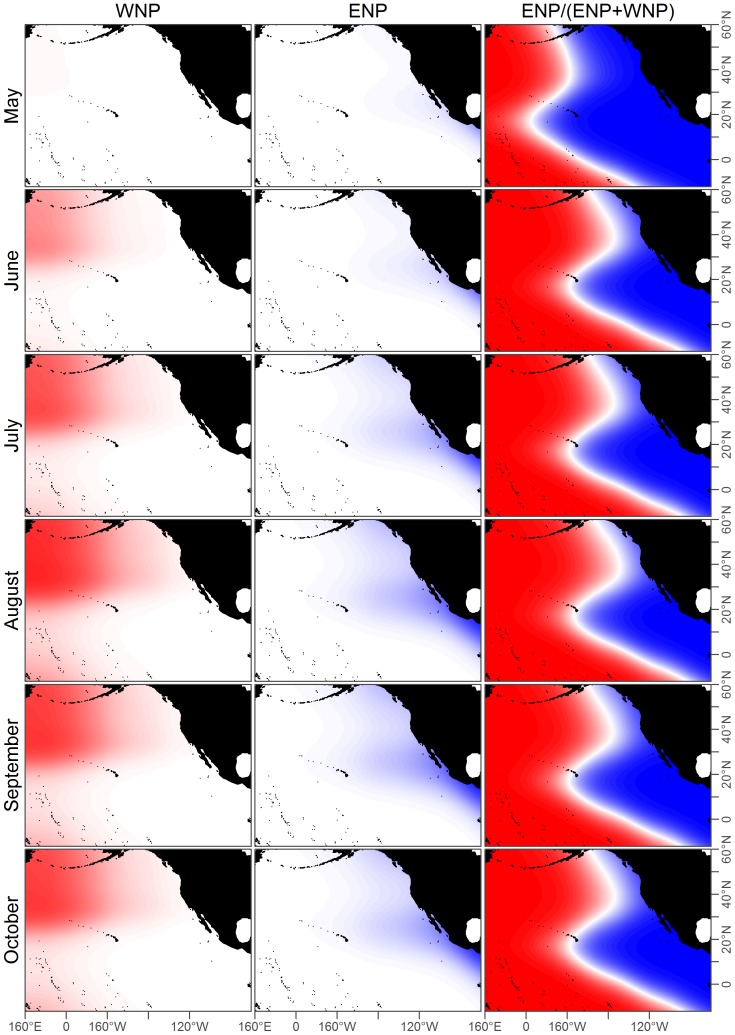
Fits for ENP acoustic, WNP acoustic, and base case prediction models for May-October. These models are fit to the acoustic data in Figures 4 and 5. The third column shows the base case prediction model which is the proportion of ENP to WNP calls, such that red areas correspond to predicted WNP occurrence and blue areas to ENP. The white line denotes where the model predicts an equal chance of observing an ENP or WNP blue whale.

**Figure 7:**
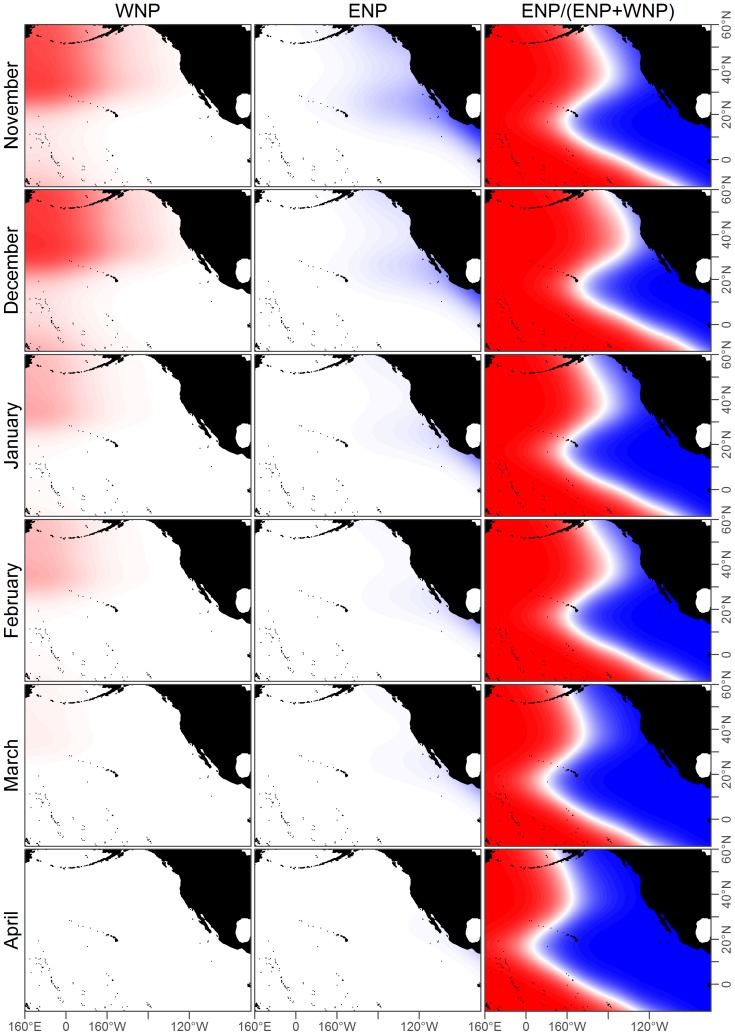
Fits for ENP acoustic, WNP acoustic, and base case prediction models for November-April. These models are fit to the acoustic data in Figures 4 and 5. The third column shows the base case prediction model which is the proportion of ENP to WNP calls, such that red areas correspond to predicted WNP occurrence and blue areas to ENP. The white line denotes where the model predicts an equal chance of observing an ENP or WNP blue whale.

AICc provided clear justification for the more complex beta-binomial structure for acoustic models as well as additive terms on parameters 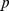 and 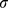 in both the ENP and WNP acoustic models (Table 3). Therefore the full acoustic models were selected for both ENP and WNP populations and used throughout the rest of the analysis. These model predictions depended on the month, but generally classified parts of the Gulf of Alaska, the west coast of the US, and the eastern tropical Pacific as being predominantly ENP (Figures 6 and 7).

**Table 3:** Model selection results.

Distribution	Probability 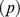	Over-dispersion 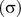	df^1^	ENP 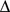 AICc	WNP 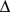 AICc
Beta-Binomial	Additive	Additive	40	0	0
Beta-Binomial	Additive	Linear	34	19	10
Beta-Binomial	Additive	Constant	21	33	36
Beta-Binomial	Linear	Linear	28	103	54
Binomial	Additive	N/A	20	24,063	12,675
Binomial	Linear	N/A	14	33,457	18,319

1 degrees of freedom used in the model

Models were compared across likelihoods and parameter structures. The smallest 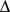
AICc indicated the model with the highest support was the most complex model. The binomial model in particular fits extremely poorly, confirming the need for an over-dispersed likelihood.

The algorithm of drawing random catch series and bootstrapping was run to produce 1000 ENP catch series. Some acoustic models failed to converged (1.73% ENP and 2.91% WNP), and since both acoustic models are needed for the prediction model, only cases where both converged for the same bootstrap sample could be used. A total of 1099 iterations of the algorithm were needed to arrive at 1000 successful ENP catch series.

**Figure 8:**
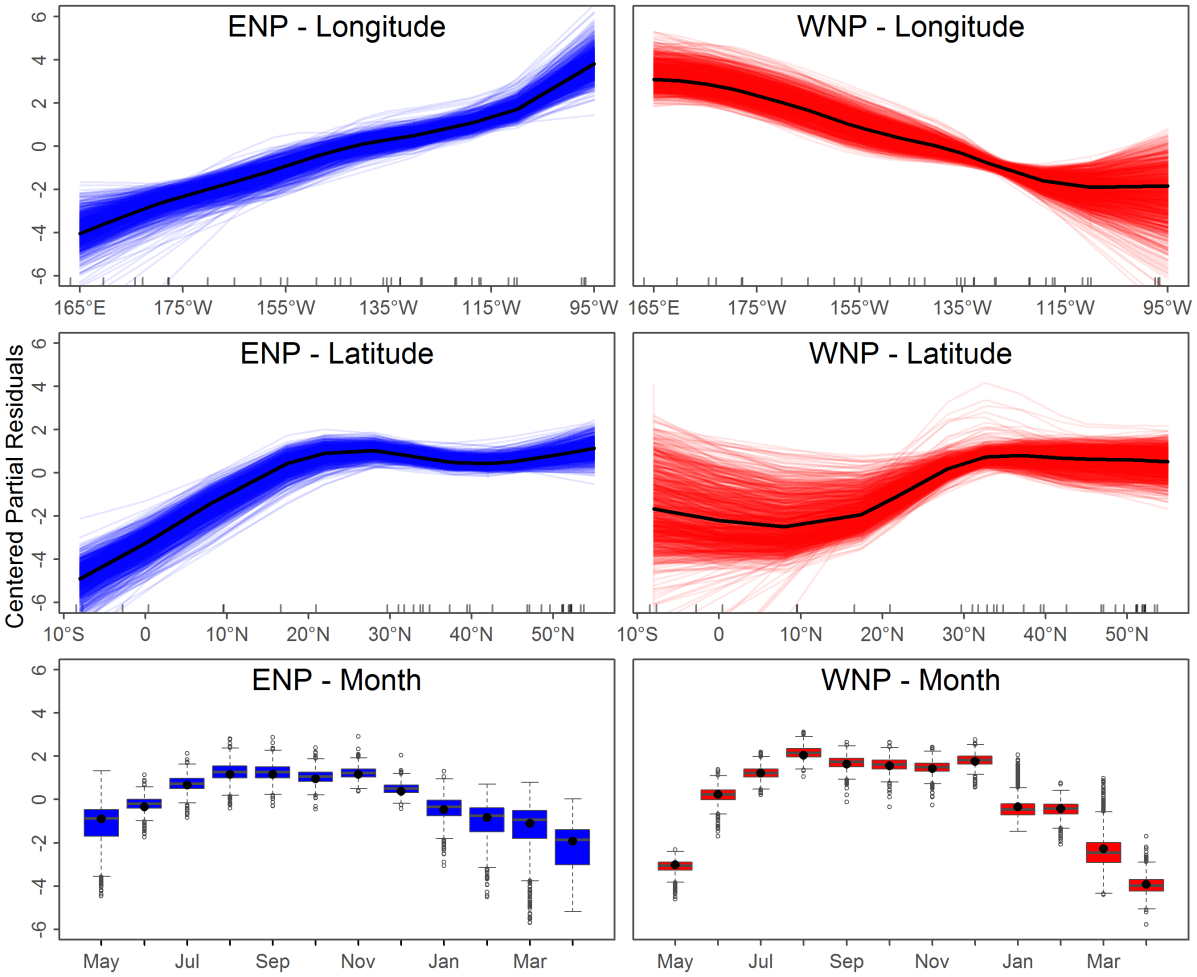
Fits of the independent variables for the probability of observing a song call (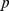
parameter). The original model (black lines) and subsequent bootstrapped models (thin colored lines) are shown. Each panel shows the relationship between the dependent and independent variable after all other independent variables have been accounted for (i.e. the centered partial residuals). Higher relative values indicate a higher probability of observing a call. For longitude and latitude, 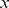-axis tick marks show positions of the observed hydrophones with a small amount of noise added to prevent overplotting. See text for further discussion.

**Figure 9:**
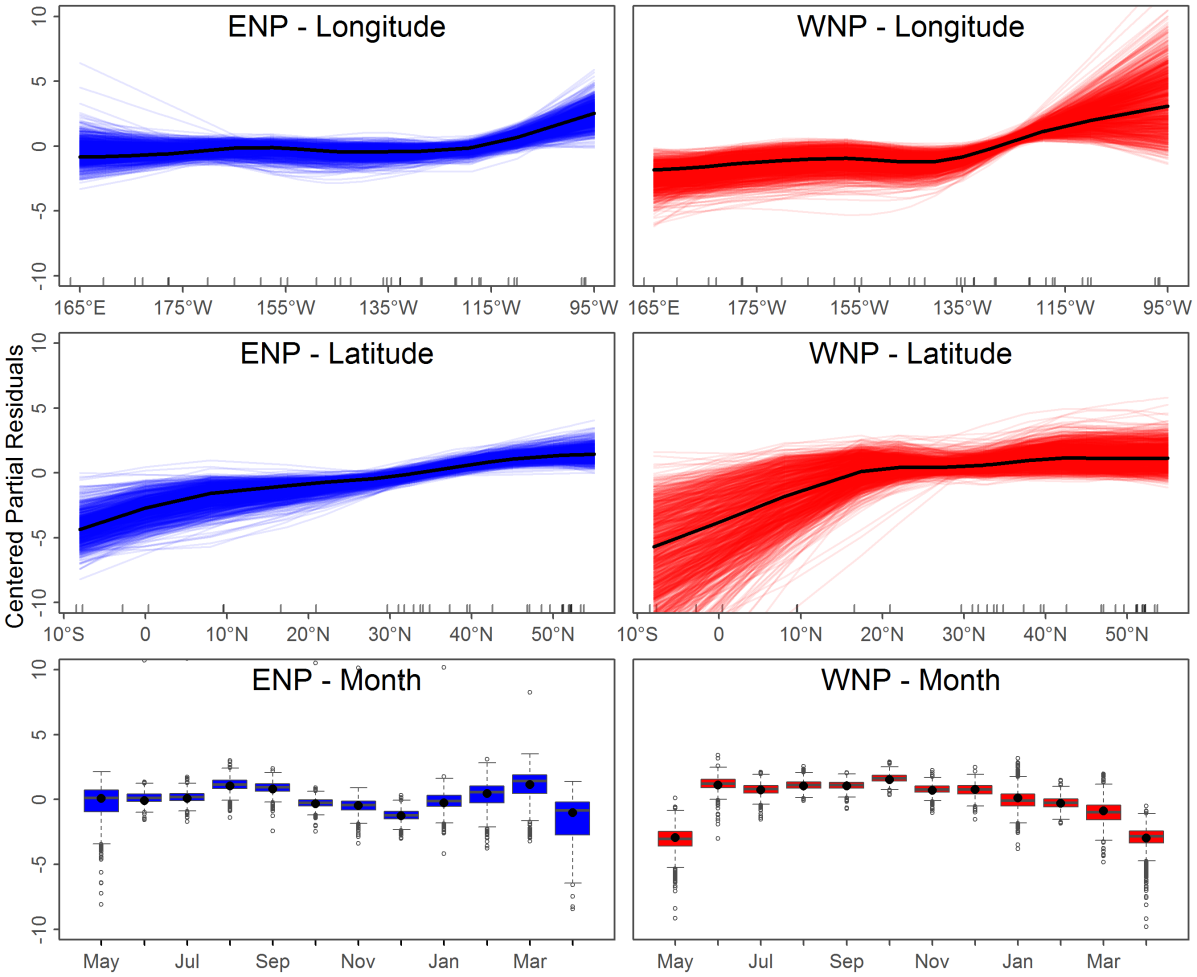
As for **Figure 8** except the results pertain to the over-dispersion parameter 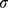. Higher relative values indicate a higher level of variance expected in repeated observations.

GAMLSS model additive fits are interpreted by plotting the smoothers fit to “partial residuals” which have an arbitrary absolute scale and instead are judged via the relative change within a single independent variable (i.e. longitude, latitude, and month). In this case, increasing partial residual values indicated that the model predicted higher call rates for 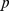, or higher variance for 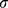. For instance, both populations showed increased calling in the summer and fall months, ENP calls decreased from east to west, and WNP calls increased for east to west (Figures 8 and 9).

**Figure 10:**
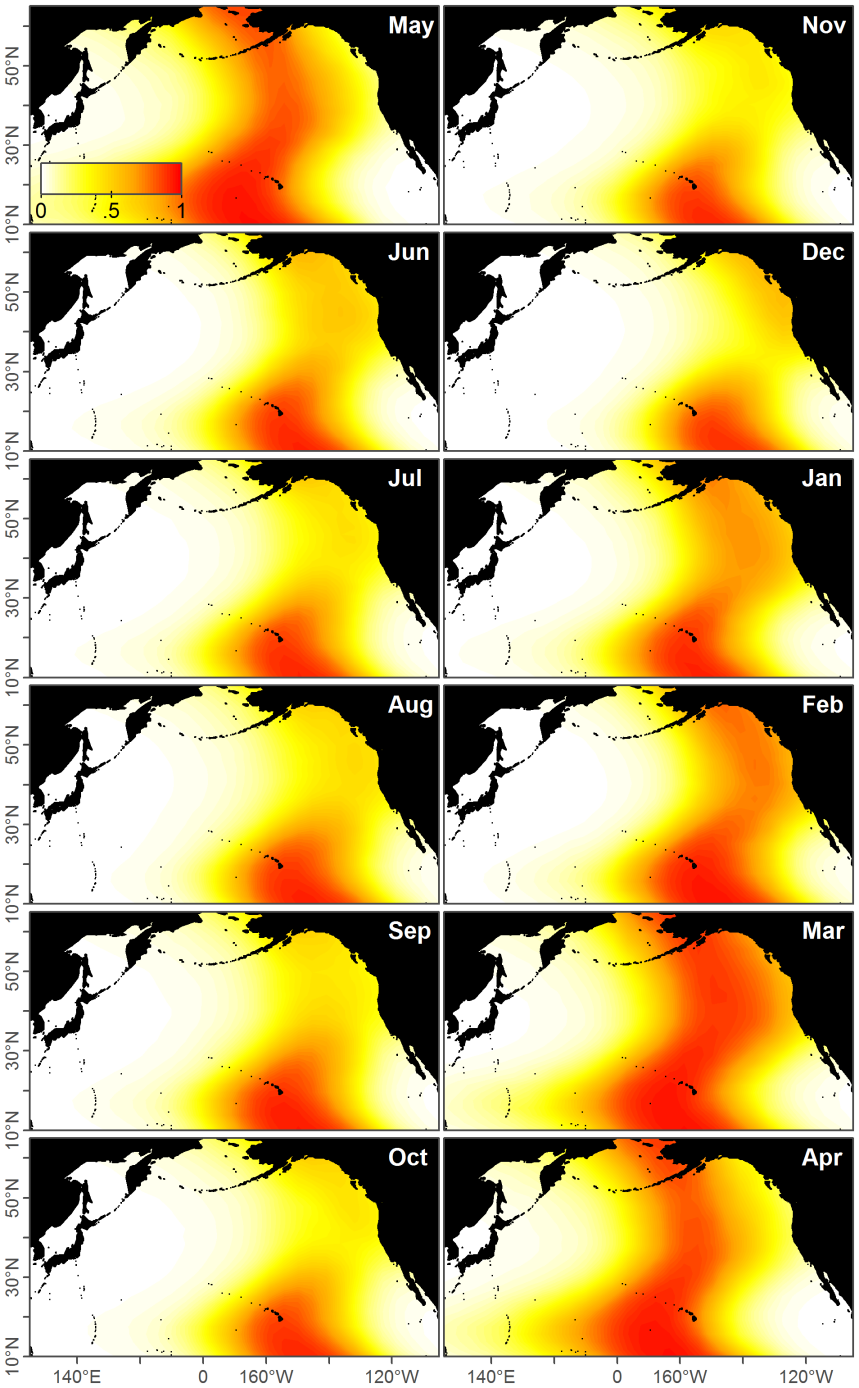
Maps showing the statistical uncertainty arising from bootstrapping. Uncertainty in the base case probability of a catch being from the ENP population quantified using bootstrapping (i.e. resampling the acoustic data with replacement and refitting the models). The 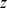-axis is the range of the predictions (95th minus 5th percentile) across all 1000 bootstrapped models. A value of 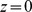 indicates all bootstrap models predict the same value, while 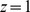 indicates some bootstrap models predict 0 and others predict 1, so that there is high uncertainty at that location. There is higher uncertainty where overlap between the two populations exists (e.g. Gulf of Alaska) or there is limited data (e.g. Hawaii).

The variance in the parameters' additive fits across bootstrapped models provided a measure of statistical uncertainty. There was more uncertainty in the months with fewer observed calls and regions with ENP and WNP overlap and limited hours with observed calls (Figure 10). For this study, the uncertainty only impacts the results where it coincides with historical catches. Thus, while there is high uncertainty around Hawaii for all months, this has little influence on the results and more acoustic data here would likely have a minimal impact. The statistical uncertainty in the GOA in the summer months (the peak of catches) has the greatest influence on the uncertainty in the results.

### Catch Estimates

**Figure 11:**
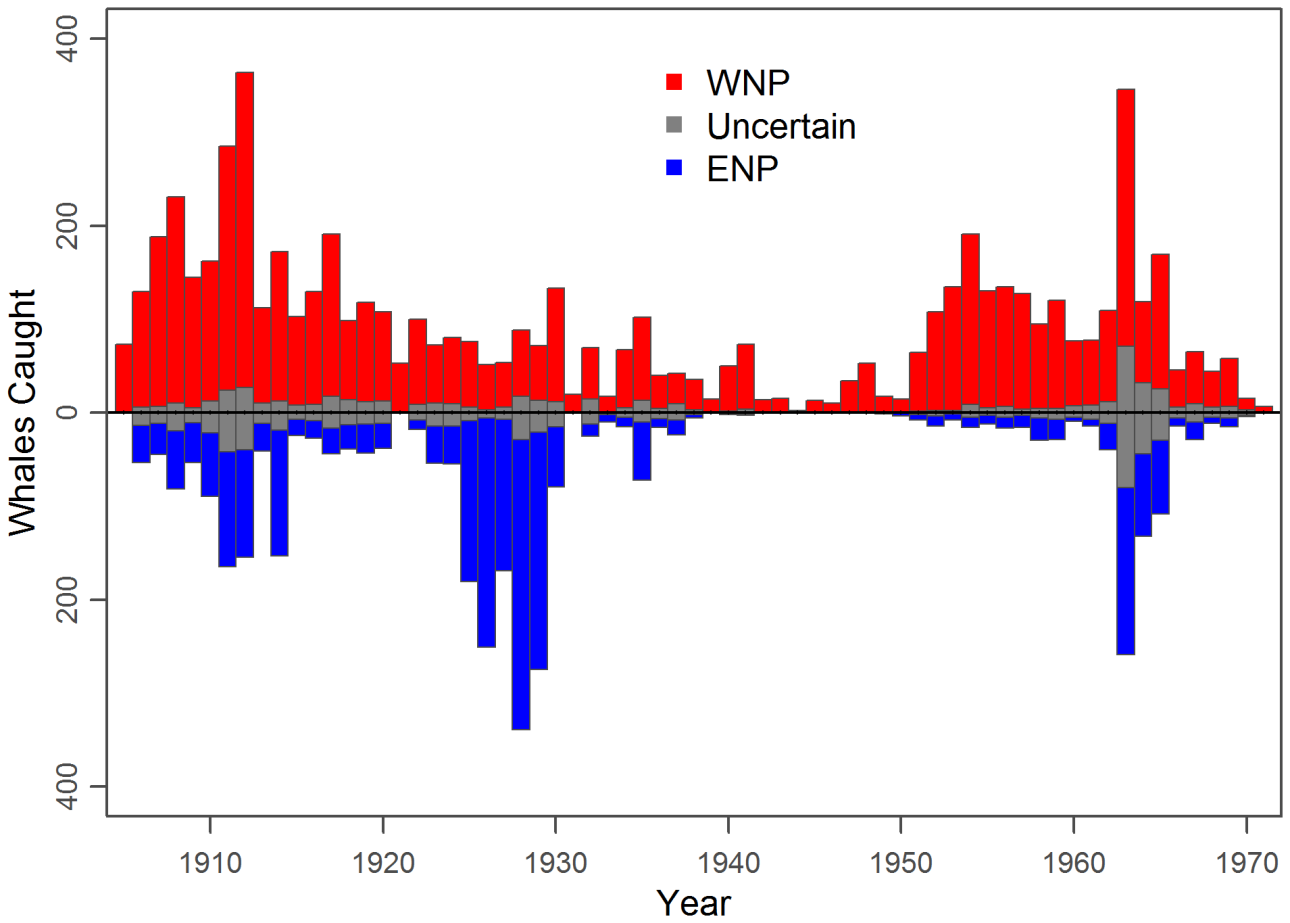
Estimated annual catches of ENP and WNP blue whales for the base case. Grey values contain 95% of the population uncertainty, and if above the line are more likely to be WNP, below the line more likely to be ENP. The uncertainty in the 1960s is caused by the unreported Soviet catches with high location uncertainty.

**Figure 12:**
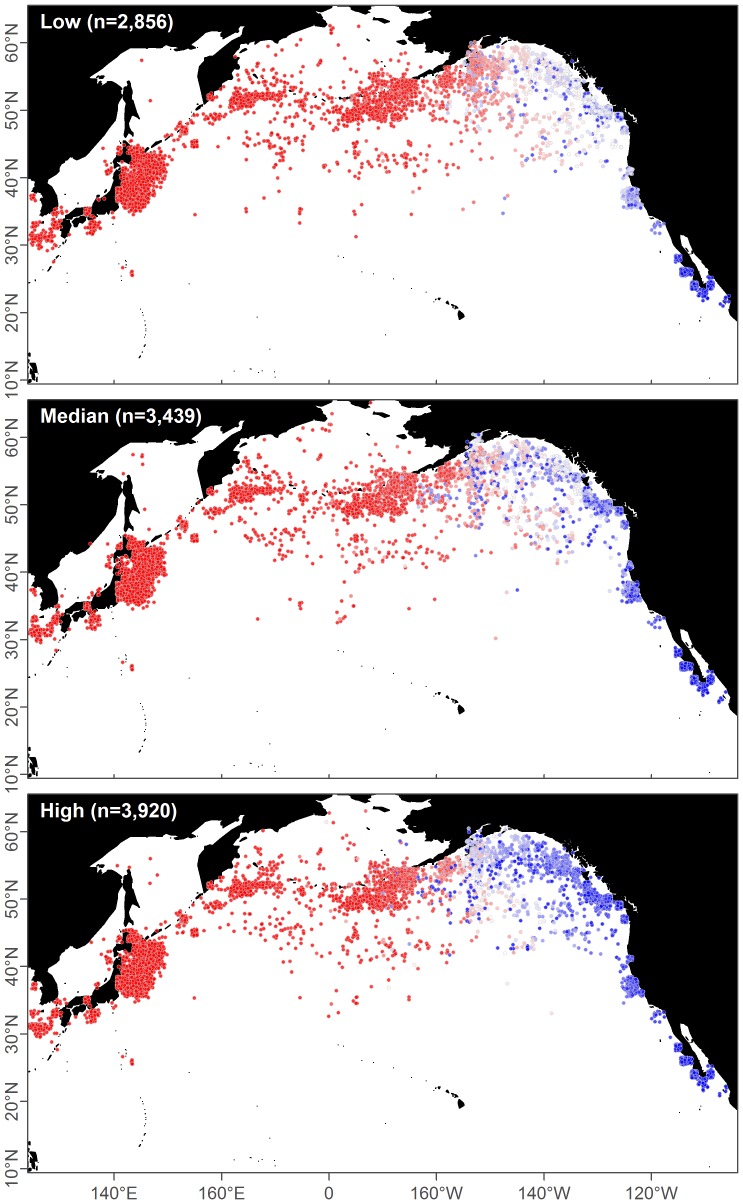
Example model predictions. Catches of ENP (blue) and WNP (red) blue whales for three realizations chosen to represent low, median, and high total ENP catches. Catch positions are plotted with a small amount of noise to prevent overplotting, and are different between the three cases because they are drawn randomly from potential locations. Nearby catches can have different predictions (colors) because months are aggregated.

Each successful iteration of the algorithm provided distinct series of ENP catches and WNP catches, which were then aggregated by year (Tables 4–5). Most catches were taken in the early part of the century (1905–1930), with another spike in the 1950s and 1960s from Soviet pelagic whaling (Figure 11). Likewise, the uncertainty was highest during the early part of the 20^th^ century when many catches were reported without location and/or month, and in the 1960s due to misreported Soviet catches which contained large catch uncertainty. Examples of specific ENP catch series demonstrate the general approach implemented here, including catch and statistical uncertainty predicted by the model (Figure 12). The catch uncertainty is reflected by the difference in locations of the catches between cases, and the statistical uncertainty by the differences in the region of uncertainty between cases.

**Table 4:** Base case ENP catches by year showing the median as well as lower and upper 95% interval from the 1000 realizations.

Year	2.5%	Median	97.5%	Year	2.5%	Median	97.5%
1905	1	1	1	1939	0	0	1
1906	40	54	60	1940	1	2	3
1907	33	45	51	1941	1	3	6
1908	62	82	92	1942	1	1	1
1909	42	53	59	1943	0	0	0
1910	68	90	102	1944	0	0	0
1911	123	165	189	1945	0	0	0
1912	115	155	182	1946	0	0	1
1913	30	41	51	1947	0	0	1
1914	134	153	165	1948	0	0	1
1915	17	25	33	1949	1	2	2
1916	19	27	36	1950	3	3	4
1917	27	44	61	1951	5	8	9
1918	26	39	52	1952	11	14	16
1919	31	43	55	1953	6	7	10
1920	27	38	50	1954	10	15	24
1921	0	0	0	1955	9	12	17
1922	10	18	27	1956	12	17	23
1923	39	54	64	1957	13	16	20
1924	41	54	64	1958	24	30	34
1925	172	181	187	1959	22	29	33
1926	245	251	253	1960	4	9	17
1927	162	169	175	1961	8	15	22
1928	310	339	357	1962	28	40	51
1929	254	275	288	1963	179	259	330
1930	64	79	92	1964	89	132	165
1931	0	0	0	1965	79	108	134
1932	13	25	40	1966	9	15	21
1933	8	10	11	1967	19	29	38
1934	10	15	20	1968	6	12	17
1935	62	72	85	1969	10	15	21
1936	9	16	20	1970	1	4	7
1937	16	24	34	1971	0	0	0
1938	4	5	8	Total	2,765	3,439	3,993

These catches do not include uncertainty in 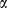.

**Table 5:** Base case WNP catches by year showing the median as well as lower and upper 95% interval from the 1000 realizations.

Year	2.5%	Median	97.5%	Year	2.5%	Median	97.5%
1905	73	73	73	1939	14	15	15
1906	123	129	143	1940	49	50	51
1907	182	188	200	1941	70	73	75
1908	221	231	251	1942	14	14	14
1909	139	145	156	1943	15	15	15
1910	150	162	184	1944	2	2	2
1911	261	285	327	1945	13	13	13
1912	337	364	404	1946	9	10	10
1913	103	113	124	1947	33	34	34
1914	160	172	191	1948	52	53	53
1915	95	103	111	1949	17	17	18
1916	121	130	138	1950	14	15	15
1917	174	191	208	1951	63	64	67
1918	85	98	111	1952	106	108	111
1919	106	118	130	1953	132	135	136
1920	96	108	119	1954	183	192	197
1921	53	53	53	1955	125	130	133
1922	91	100	108	1956	128	134	139
1923	62	72	87	1957	123	127	130
1924	71	81	94	1958	91	95	101
1925	70	76	85	1959	116	120	127
1926	49	51	57	1960	69	77	82
1927	48	54	61	1961	70	77	84
1928	71	89	118	1962	98	109	121
1929	59	72	93	1963	275	346	426
1930	121	134	149	1964	86	119	162
1931	20	20	20	1965	144	170	199
1932	55	70	82	1966	39	45	51
1933	16	17	19	1967	56	65	75
1934	62	67	72	1968	39	44	50
1935	89	102	112	1969	52	58	63
1936	36	40	47	1970	12	15	18
1937	32	42	50	1971	7	7	7
1938	33	36	37	Total	5,780	6,334	7,008

These catches do not include uncertainty in 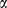.

**Figure 13:**
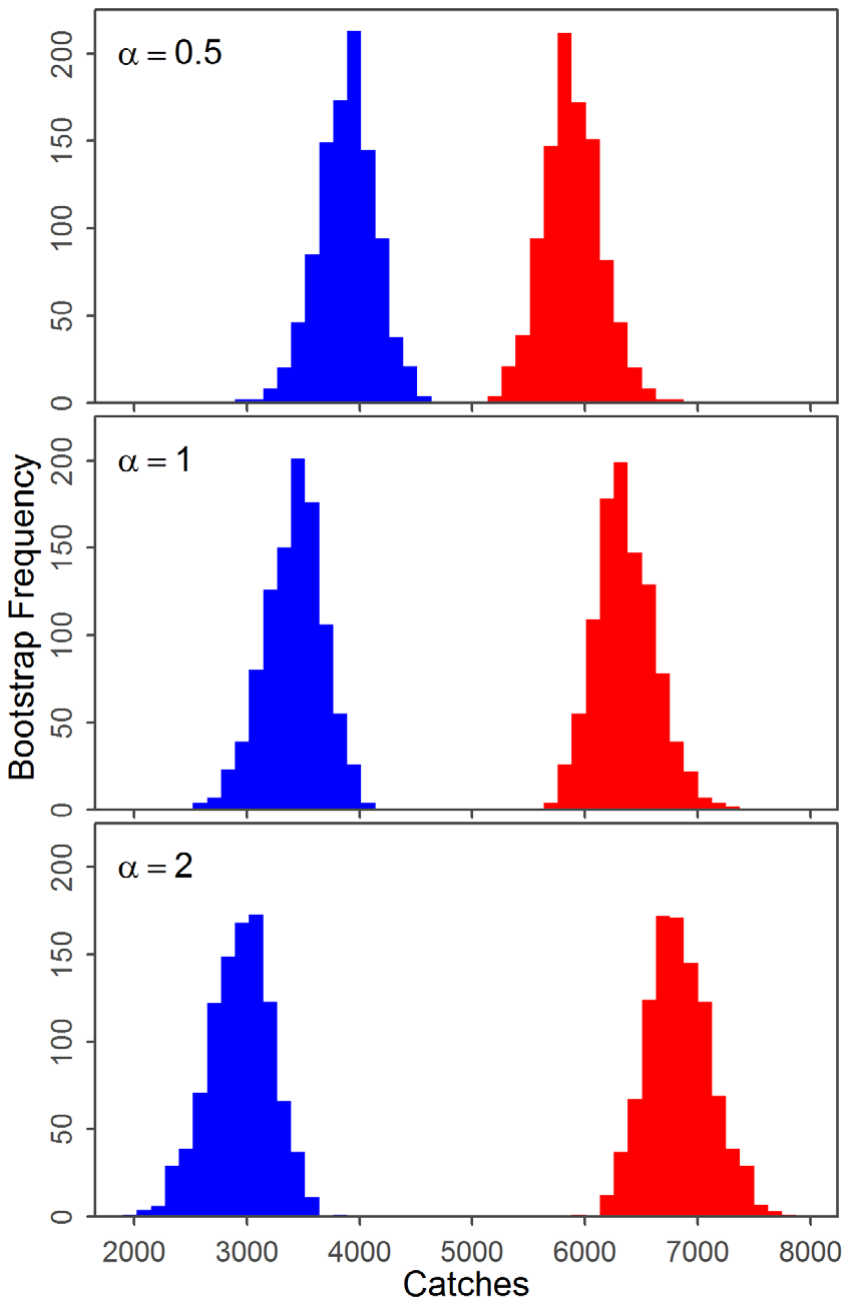
Results of the sensitivity analysis to the conversion between song call frequency and density of whales. The sensitivity to the base case assumption that 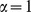 to the final results is explored with histograms of the total catches of the ENP (blue) and WNP (red) populations for three levels of 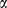. 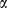 is the ratio of the unknown WNP and ENP factors which convert density of whales to song call occurrence.

Our methods allowed uncertainty to be estimated for the catch series, and incorporated into the results. The largest source of uncertainty for the base case came from bootstrapping the acoustic data. The catch uncertainty was relatively small compared to the statistical uncertainty, thus additional acoustic data has the potential to decrease the uncertainty in the results the most (Table 6). The ecological sensitivity run where 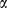 ranged uniformly between 0.5 and 2 contributed more uncertainty than catch uncertainty, but less than statistical uncertainty (Table 6). As expected a higher value of 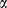 for all algorithm iterations lead to smaller estimates for the total ENP catches (Figure 13).

**Table 6:** Comparison of the sources of uncertainty in total ENP catches.

Uncertainty Source	2.5%	Median	97.5%	Cumulative % Uncertainty
Catch	3,430	3,452	3,470	3%
Catch + Statistical	2,855	3,441	3,920	70%
Catch + Statistical + Ecological	2,593	3,411	4,114	100%

Catch uncertainty arises from missing locations and dates, statistical uncertainty from song call occurrence data, and ecological from uncertainty in ecological assumptions over the range 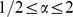. Differences in totals compared to Table 4 are due to rounding and the order of percentile calculations.

We estimate that the total number of ENP whales caught from 1905–1971 in the North Pacific was 3,441 (95% range 2,855–3,920, out of 9,773 total) based on catch and statistical uncertainty. For the sensitivity case with ecological uncertainty included, the total number of ENP blue whales caught was 3,411 (95% range 2,593–4,114). Thus, the inclusion of ecological uncertainty increases the width of the 95% interval by 32% if 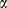 is assumed to range uniformly from 0.5 to 2. Catches of WNP blue whales totaled 6,332 (95% range 5,853–6,918) with catch and statistical uncertainty, while the case with ecological uncertainty was 6,362 (95% range 5,659–7,180).

### Model Validation

Movement of ENP whales between the ETP, California, and the Gulf of Alaska from satellite telemetry and photographic identification [13], [14] corroborated our model's prediction that ENP whales occur throughout this range (Figures 6–7).

**Figure 14:**
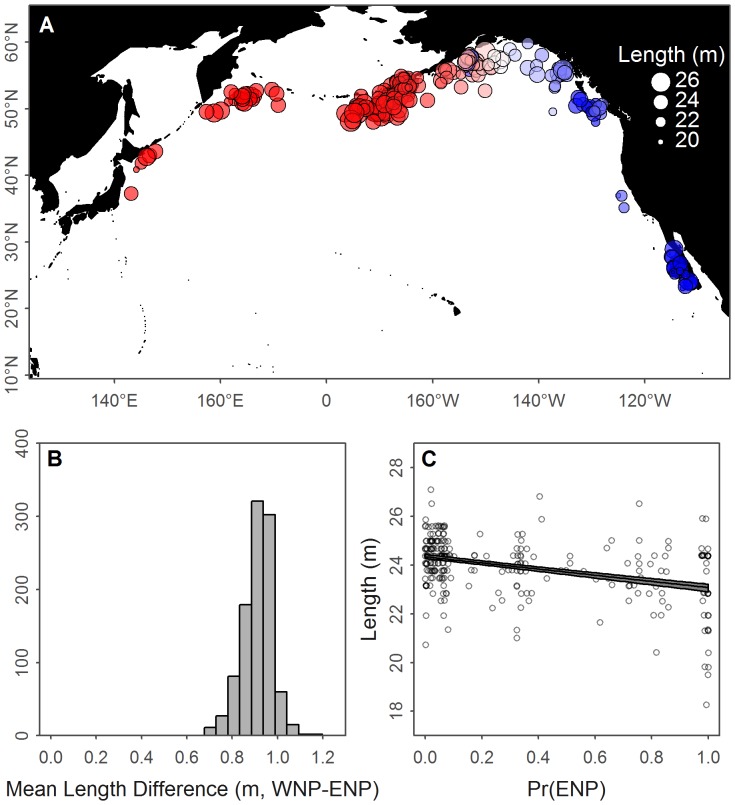
Results of the length analysis of mature female blue whales. (A) Locations, lengths, and median predicted population (color) for all years. (B) The distribution of mean length differences for all bootstrap iterations. (C) The 95% percentiles (gray polygon) and median line for all bootstrap linear models fit to the predicted population versus lengths. The circles represent the median probability of being ENP and observed lengths and are plotted with a small amount of noise to prevent overplotting.

We applied the length analysis to 258 mature females, compared to 252 from [15], which were distributed sufficiently across time and space for the statistical tests (Figure 14a). For the two-sample 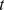-tests, the median difference in mean lengths was 0.91 m (95% range 0.76–1.03 m, Figure 14b) and for all 1000 catch scenarios this difference was highly significant (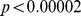). The slopes of the linear regressions were all significantly negative, demonstrating that the shorter a whale is, the more likely it from the ENP population (Figure 14c).

## Discussion

Our study is the first to split North Pacific blue whale catches by population, and was based on current day patterns in acoustic song types across the North Pacific. Our results based on acoustic calls were validated using the length data in the catches: those individuals we assigned to the ENP were shorter (on average by 0.91 m) and these differences were highly significant. We attributed roughly 35% of all blue whales catches to the ENP population, suggesting that the WNP population was likely substantially larger than the ENP population before whaling started. Current abundance estimates are not available for any region within the WNP, so it is unclear whether the WNP blue whales are now more or less depleted than ENP blue whales.

Our results are based on the following ecological assumptions about movement patterns and acoustic behavior of the two populations. First, we assumed that the ENP and WNP populations had stable movement patterns over the entire span of the catches and acoustic recordings (1905–2004), despite intensive whaling and varying oceanographic and biological conditions. If ample acoustic data were available across many years, the effect of year could be quantified within the modeling framework provided here, but the acoustic data were collected in different time periods (Table 2), precluding such an analysis in this study. High maternal site fidelity in the Gulf of California has been shown for the ENP population, supporting the idea of spatial continuity in a breeding and calving region [16], but another study speculates a range shift into the Gulf of Alaska due to changes in abundance or oceanographic conditions [14]. It seems reasonable that slight changes in ranges for the ENP, particularly in the GOA are possible, but highly unlikely that they formerly occurred in the western North Pacific. For the WNP there is no information with which to judge how many populations there are, or their spatial fidelity.

Second, we assumed that the ratio of calling rates was proportional to the ratio of densities of the two populations across time and space, but cannot directly test the accuracy of this hypothesis. The relationship between singing and density is likely complex, especially if it is related to breeding. One study suggests that ENP blue whales arriving at feeding grounds may delay singing for weeks until adequate food has been obtained [12] and another in the same area found the proportion of whales singing increased during the feeding season [33]. Thus the relationship between density and ENP singing is weak for part of the feeding season, though this short-term effect is mitigated to some degree by binning the data into months. Little is known about the seasonal behavior of ENP blue whales, and none about WNP whales, so we could not explicitly include this in our analysis. The assumption may also be incorrect if there were a difference in the sex ratios or sex-specific mixing between populations. The historical catch records clearly indicated that both sexes were caught in similar ratios throughout the region modeled, so this is not believed to be a major issue in the analysis. In addition, the two populations could vocalize at different rates. Since they have similar morphologies, call types, seasonal pattern of call production, and life history strategies it is unlikely that the behavioral context of call production differs greatly between populations.

The veracity of the ecological assumptions discussed here was, and may remain, unknown. However, when we tested the influence of this factor in a sensitivity test allowing 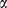 to range from 0.5 to 2.0 (a fourfold range), the 95% confidence interval in the total ENP catches widened by only 32%. This suggests that the results are fairly robust to these assumptions. Similarly, the relatively small impact from catch uncertainty suggests the simplifying assumptions made, such as using reported locations without error and ignoring the selective preference of expeditions, likely had a minimal impact on the results.

Although the focus of this study was ENP blue whales, by examining all catch and acoustic data, new information about WNP blue whale exploitation emerged. We estimated 6,332 (95% range 5,852–6,918) or roughly 65% of blue whale catches in the North Pacific were likely from the WNP population. Unlike ENP blue whales the current abundance, status, and geographic range of these whales remains unknown. At present there is no dedicated effort to study WNP whales and acoustic data provide the only information as to when and where these blue whales are found in the North Pacific.

We incorporated uncertainty into how the catches were split between populations, but there still may be missing catches, particularly off Japan in the early part of the century where many catches were not identified to species in 1905–1909. Future studies utilizing WNP catch data should visit those catches in further detail. Missing catches may also exist for the Soviet pelagic expeditions in the 1960s and 1970s, which would affect the results here, but is likely that most of these have been recovered and revised already and few more exist [24].

This study provides a new framework for differentiating spatial occurrence of populations of cetaceans using their distinct call types using data that are relatively easy to collect in comparison to broad scale sighting surveys, photo identification, and genetic biopsy sampling efforts. In doing so we provide the first estimate of catches for ENP blue whales that is based on data and includes uncertainty. Future work should combine these results with current abundance estimates to formally assess the recovery of the population and the potential risk of continued anthropogenic mortalities.

## References

[1] Rice DW (1998) Marine mammals of the world: systematics and distribution: Society of Marine Mammalogy.

[2] Reilly SB, Bannister JL, Best PB, Brown M, RL BJ, et al. (2008) *Balaenoptera musculus*. In: IUCN 2012. IUCN Red List of Threatened Species. Version 2012.2.

[3] Carretta JV, Oleson E, Weller DW, Lang AR, Forney KA, et al. (2012) U.S. Pacific marine mammal stock assessments. NOAA Technical Memorandum NOAA-TM-NMFS-SWFSC-504.

[4] Stafford KM, Nieukirk SL, Fox CG (1999) An acoustic link between blue whales in the eastern tropical Pacific and the northeast Pacific. Marine Mammal Science 15: 1258–1268.

[5] Goldbogen JA, Calambokidis J, Oleson E, Potvin J, Pyenson ND, et al (2011) Mechanics, hydrodynamics and energetics of blue whale lunge feeding: efficiency dependence on krill density. Journal of Experimental Biology 214: 698–699. 10.1242/jeb.048157 21147977

[6] Oleson EM, Calambokidis J, Burgess WC, McDonald MA, LeDuc CA, et al (2007) Behavioral context of call production by eastern North Pacific blue whales. Marine Ecology-Progress Series 330: 269–284.

[7] Melcon ML, Cummins AJ, Kerosky SM, Roche LK, Wiggins SM, et al (2012) Blue whales respond to anthropogenic noise. PLoS One 7: e32681. 22393434 10.1371/journal.pone.0032681 PMC3290562

[8] Stafford KM, Nieukirk SL, Fox CG (2001) Geographic and seasonal variation of blue whale calls in the North Pacific. Journal of Cetacean Research and Management 3: 65–76.

[9] Stafford KM (2003) Two types of blue whale calls recorded in the Gulf of Alaska. Marine Mammal Science 19: 682–693.

[10] McDonald MA, Hildebrand JA, Mesnick SL (2006) Biogeographic characterization of blue whale song worldwide: using song to identify populations. Journal of Cetacean Research and Management 8: 55–65.

[11] Rivers JA (1997) Blue whale, *Balaenoptera musculus*, vocalizations from the waters off central California. Marine Mammal Science 13: 186–195.

[12] Oleson EM, Wiggins SM, Hildebrand JA (2007) Temporal separation of blue whale call types on a southern California feeding ground. Animal Behaviour 74: 881–894.

[13] Bailey H, Mate BR, Palacios DM, Irvine L, Bograd SJ, et al (2010) Behavioural estimation of blue whale movements in the Northeast Pacific from state-space model analysis of satellite tracks. Endangered Species Research 10: 93–106.

[14] Calambokidis J, Barlow J, Ford JKB, Chandler TE, Douglas AB (2009) Insights into the population structure of blue whales in the Eastern North Pacific from recent sightings and photographic identification. Marine Mammal Science 25: 816–832.

[15] Gilpatrick Jr JW, Perryman WL (2008) Geographic variation in external morphology of North Pacific and Southern Hemisphere blue whales (*Balaenoptera musculus*). Journal of Cetacean Research and Management 10: 9–21.

[16] Costa-Urrutia P, Sanvito S, Victoria-Cota N, Enríquez-Paredes L, Gendron D (2013) Fine-scale population structure of blue whale wintering aggregations in the Gulf of California. PLoS One 8: e58315. 23505485 10.1371/journal.pone.0058315 PMC3591444

[17] Reeves RR, Clapham PJ, Brownell Jr RL, Silber GK (1998) Recovery plan for the blue whale (*Balaenoptera musculus*). Publications, Agences and Staff of the U.S. Department of Commerce. Paper 118.

[18] Baker CS, Clapham PJ (2004) Modelling the past and future of whales and whaling. Trends in Ecology & Evolution 19: 365–371. 16701287 10.1016/j.tree.2004.05.005

[19] Calambokidis J, Falcone E, Douglas A, Schlender L, Huggins J (2009) Photographic identification of humpback and blue whales off the U.S. West Coast: results and updated abundance estimates from 2008 field season. Final report for contract AB133F08SE2786. Southwest Fisheries Science Center.

[20] Calambokidis J, Barlow J (2004) Abundance of blue and humpback whales in the eastern North Pacific estimated by capture-recapture and line-transect methods. Marine Mammal Science 20: 63–85.

[21] Allison C (2011) IWC summary catch database Version 5.2.

[22] Allison C (2011) IWC individual catch database Version 5.2.

[23] Yablokov AV (1994) Validity of whaling data. Nature 367: 108–108.

[24] Ivashchenko YV, Clapham PJ, Brownell Jr RL (2013) Soviet catches of whales in the North Pacific: revised totals. Journal of Cetacean Research and Management 13(1): 59–71.

[25] R Core Team (2012) R: A language and environment for statistical computing. 1.7.0 ed. Vienna, Austria: R Foundation for Statistical Computing.

[26] Stafford KM, Nieukirk SL, Fox CG (1999) Low-frequency whale sounds recorded on hydrophones moored in the eastern tropical Pacific. Journal of the Acoustical Society of America 106: 3687–3698. 10615707 10.1121/1.428220

[27] McDonald MA, Fox CG (1999) Passive acoustic methods applied to fin whale population density estimation. Journal of the Acoustical Society of America 105: 2643–2651.

[28] Gregr EJ, Trites AW (2001) Predictions of critical habitat for five whale species in the waters of coastal British Columbia. Canadian Journal of Fisheries and Aquatic Sciences 58: 1265–1285.

[29] Wood SN (2006) Generalized additive models: an introduction with R: Chapman & Hall.

[30] Rigby RA, Stasinopoulos DM (2005) Generalized additive models for location, scale and shape. Journal of the Royal Statistical Society Series C-Applied Statistics 54: 507–544.

[31] Burnham KP, Anderson DR (2002) Model selection and multi-model inference. Berlin, Heidelberg, New York: Springer.

[32] Efron B, Tibshirani R (1993) An introduction to the bootstrap: Chapman & Hall/CRC.

[33] Oleson EM, Calambokidis J, Barlow J, Hildebrand JA (2007) Blue whale visual and acoustic encounter rates in the southern California bight. Marine Mammal Science 23: 574–597.

